# Generation of Human-Induced Pluripotent Stem Cell-Derived Functional Enterocyte-Like Cells for Pharmacokinetic Studies

**DOI:** 10.1016/j.stemcr.2020.12.017

**Published:** 2021-01-28

**Authors:** Shinpei Yoshida, Takayuki Honjo, Keita Iino, Ryunosuke Ishibe, Sylvia Leo, Tomoka Shimada, Teruhiko Watanabe, Masaya Ishikawa, Kazuya Maeda, Hiroyuki Kusuhara, Nobuaki Shiraki, Shoen Kume

**Affiliations:** 1 School of Life Science and Technology, Tokyo Institute of Technology, 4259-B-25 Nagatsuta-cho, Midori-ku, Yokohama, Kanagawa 226-8501, Japan; 2 Drug Metabolism & Pharmacokinetics, Research Laboratory for Development, Shionogi & Co., Ltd., 1-1, Futabacho 3-chome, Toyonaka, Osaka 561-0825, Japan; 3 Analytical Chemistry & Technology, Shionogi TechnoAdvance Research Co., Ltd., 1-1, Futabacho 3-chome, Toyonaka, Osaka 561-0825, Japan; 4 Isehara Research Laboratory, Technology and Development Division, Kanto Chemical Co. Inc., 21 Suzukawa, Isehara, Kanagawa 259-1146, Japan; 5 Laboratory of Molecular Pharmacokinetics, Graduate School of Pharmaceutical Sciences, The University of Tokyo, 7-3-1 Hongo, Bunkyo-ku, Tokyo 113-0033, Japan

**Keywords:** *in vitro* differentiation, induced pluripotent stem cells, intestine, drug development, pharmacokinetics, enterocyte, human model

## Abstract

We aimed to establish an *in vitro* differentiation procedure to generate matured small intestinal cells mimicking human small intestine from human-induced pluripotent stem cells (iPSCs). We previously reported the efficient generation of CDX2-expressing intestinal progenitor cells from embryonic stem cells (ESCs) using 6-bromoindirubin-3′-oxime (BIO) and (3,5-difluorophenylacetyl)-L-alanyl-L-2-phenylglycine *tert*-butyl ester (DAPT) to treat definitive endodermal cells. Here, we demonstrate the generation of enterocyte-like cells by culturing human iPSC-derived intestinal progenitor cells on a collagen vitrigel membrane (CVM) and treating cells with a simple maturation medium containing BIO, DMSO, dexamethasone, and activated vitamin D3. Functional tests further confirmed that these iPSC-derived enterocyte-like cells exhibit P-gp- and BCRP-mediated efflux and cytochrome P450 3A4 (CYP3A4)-mediated metabolism. We concluded that hiPS cell-derived enterocyte-like cells can be used as a model for the evaluation of drug transport and metabolism studies in the human small intestine.

## Highlights

• hiPSC-derived intestinal progenitors are differentiated into enterocytes
• The collagen vitrigel membrane supports hiPSCs to mature into functional enterocytes
• iPSC-derived enterocytes show efflux transporter and metabolizing enzyme activities
• iPSC-derived enterocytes can be used as a human small intestine model

## Teaser

We established a culture procedure to generate hiPSC-derived enterocyte-like cells that can be used as a model for the evaluation of drug transport and metabolism studies in the human small intestine.

## Introduction

The small intestine is tasked with absorbing drugs as well as nutrients, ions, and water through its enterocytes. Since membrane permeability and metabolism in the enterocytes determine the bioavailability of drugs, their impact on the oral absorption of novel chemicals is routinely assessed during the development of oral drugs using *in vitro* and animal studies. The human colon cancer cell line Caco-2 is widely used as an *in vitro* model of the intestinal epithelium (Sambuy et al., 2005). Caco-2 cells form a tight monolayer and show drug uptake/efflux mediated by some transporter isoforms such as P-glycoprotein (P-gp), but the absolute expression levels of metabolic enzymes and transporters are often different from those in the intact intestinal tissue (Sun et al., 2002). Particularly, cytochrome P450 3A4 (CYP3A4) is recognized as a critical element for the drug metabolism in the intestine since CYP3A4 is most abundantly expressed in the small intestine among CYP isoforms and about half of the approved drugs are metabolized by CYP3A4. Previous reports indicated the clinical significance of CYP3A4 in the suppression of intestinal absorption of various drugs; however, Caco-2 cells lack CYP3A4 expression and thus cannot be used for the evaluation of the intestinal availability of drugs. Furthermore, Caco-2 cells also exhibit cell line-to-cell line differences in their properties (Hayeshi et al., 2008). Therefore, a more appropriate *in vitro* model system for evaluating intestinal absorption of compounds in humans is needed.

Human embryonic stem cells (hESCs) and induced pluripotent stem cells (hiPSCs) (Takahashi et al., 2007) have the potential to differentiate and give rise to all types of cells from three germ layers, then to specific cell types upon exposure to the corresponding growth factors. Recent studies, including ours, have demonstrated the differentiation of ESCs and iPSCs into the definitive endoderm and its derivative organs, such as the pancreas, liver, and the intestine.

The intestinal epithelium is the most rapidly self-renewing tissue, thanks to the presence of intestinal stem cells (ISCs). ISCs are found in the crypts and give rise to the differentiated cell types: the absorptive cells of the enterocytes and secretory cell types such as goblet cells, enteroendocrine cells, and Paneth cells (Nakamura et al., 2007; Sato and Clevers, 2013). Mutant mice studies have identified several genes and factors necessary for the maintenance and regulation of intestinal stem cell proliferation and differentiation, including Wnt/β-catenin and Notch signaling (Chiba, 2006). ISCs express a leucine-rich orphan G-protein-coupled receptor (LGR5) (Barker et al., 2007), which is a Wnt signaling receptor that mediates Wnt/β-catenin signaling upon the binding of its ligand R-spondin1. Single sorted Lgr5+ cells have been previously found to form organoids and expand over long periods in a Matrigel-based culture supplemented with epidermal growth factor (EGF), Noggin, and R-spondin1 (Sato et al., 2009). In another study, an optimized system for the cultured mouse and human colonic epithelium was created by supplementation with Wnt3a, EGF, Noggin, R-spondin1, nicotinamide, and A83-01 (an inhibitor for transforming growth factor β [TGFβ] type I receptor kinase, also known as activin like kinase 5 [ALK5]) (Sato et al., 2011). The organoid culture system for the ISCs is used to induce the differentiation of hiPSCs into intestinal cells. hiPSCs were first differentiated into definitive endoderm by activin, followed by culturing in Matrigel supplemented with high concentrations of fibroblast growth factor 4 (FGF4) and Wnt3A to induce Caudal-related homeobox transcription factor 2 (CDX2)-expressing mid/hindgut lineage before transferring into the above three-dimensional organoid culture system (Spence et al., 2011). After the prolonged culture of these iPSC-derived intestinal cells and their transplantation into mouse kidney capsules, the hiPSC-derived cells were further matured into differentiated cell types 6 weeks after their transplantation (Watson et al., 2014).

In addition to the three-dimensional culture system, deriving intestinal epithelial cells in a two-dimensional monolayer culture has been attempted. FGF4 and Wnt3A were reported to posteriorize the endoderm into CDX2-positive intestinal cells (Ameri et al., 2010). Our group reported a two-dimensional procedure for intestinal epithelial differentiation from mouse and human ESCs. After definitive endoderm (DE) differentiation, 6-bromoindirubin-3′-oxime (BIO), a glycogen synthase kinase (GSK)-3β inhibitor, and (3,5-difluorophenylacetyl)-L-alanyl-L-2-phenylglycine *tert*-butyl ester (DAPT), a γ-secretase inhibitor, synergistically induced CDX2-expressing posterior definitive endodermal cells, which then differentiated into four mature intestinal cell types, namely enterocytes, goblet cells, enteroendocrine cells, and Paneth cells (Ogaki et al., 2013). After the optimization of the differentiation protocol, we reported the generation of mature cells of the enterocyte-like cells using a 16-day differentiation rapid protocol (Ogaki et al., 2015). Alternatively, the differentiation of iPSCs into intestinal cells could be promoted by the transduction of *CDX2* (Takayama et al., 2019). Treatments of the human iPSC-derived intestinal progenitor cells with small-molecular compounds mimicking the organoid culture system (Negoro et al., 2018) and epigenetic modifiers (Iwao et al., 2015; Kodama et al., 2016) promoted the differentiation into enterocyte-like cells that express transporters and metabolizing enzymes.

Here, we report on a simple culture procedure for the fabrication of enterocyte-like cells from human iPS cells. This system has promising applications in drug development via the culturing of endoderm or intestinal progenitor cells on the collagen vitrigel membrane (CVM). We previously reported that CVM can support the maturation of hiPS cells into hepatocytic cells (Nakai et al., 2019). By culturing on CVM, the hiPSC-derived endoderm or intestinal progenitor cells differentiated into enterocyte-like cells expressing a variety of drug transporters and metabolizing enzymes. The differentiated cells also exhibit the transporter-mediated drug transport and CYP3A4-mediated metabolism and would be useful for the prediction of intestinal absorption of drug candidates in the drug development process.

## Results

### Collagen vitrigel supports the differentiation of human iPSCs into intestinal enterocyte-like cells characterized by intestinal marker expression

**Figure 1:**
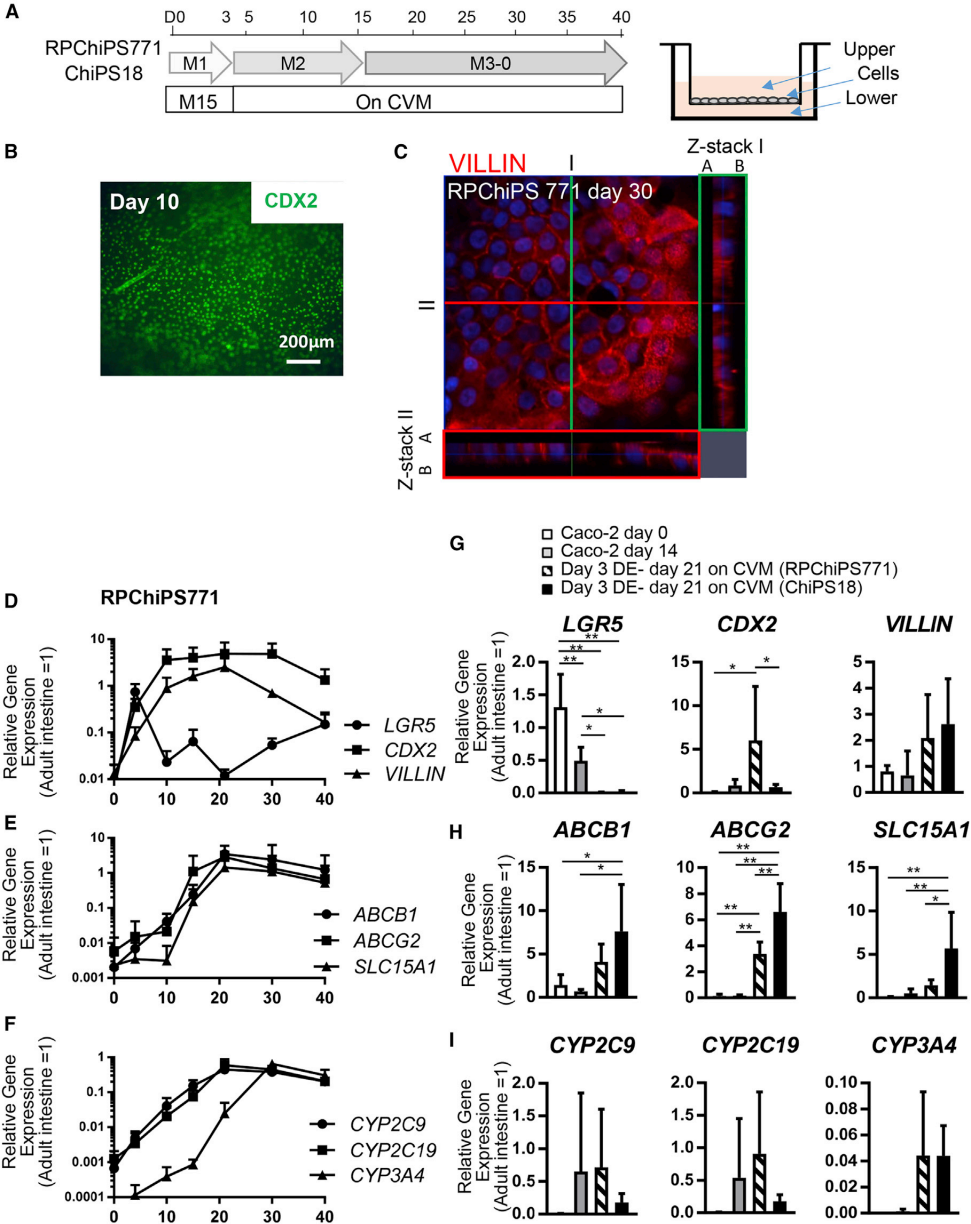
The CVM Supports the Differentiation of Human iPSCs into Intestinal Cells Expressing Molecular Markers of Transporters and CYP Enzymes (A) A schematic drawing of the differentiation procedure of hiPSCs to derive intestinal differentiation into enterocytes. (B and C) Expression of an intestinal marker CDX2 (green) on day 10 (B), VILLIN (red) expression is observed to localize in the apical side of the hiPSC-derived enterocyte-like cells. I (green line) and II (red line) depict the cross-section along which the Z-stacks are compiled and shown in the box areas (C). (D–F) Time-dependent expressions of *CDX2*, *VILLIN* (an enterocyte marker), and *LGR5* (an intestinal stem cell marker) (D). Time-dependent expressions of *ABCB1*, *ABCG2*, and *SLC15A1* (transporter) (E). Time-dependent expressions of *CYP2C9*, *CYP2C19,* and *CYP3A4* (enzyme) (F). (G–I) hiPSC-derived enterocyte-like cells expressed higher levels of *CDX2* and *VILLIN* but lower levels of *LGR5* compared with that of the Caco-2 cells (G). hiPSC-derived enterocyte-like cells expressed higher levels of *ABCB1*, *ABCG2*, and *SLC15A1* compared with those of the Caco-2 cells, respectively (H). hiPSC-derived enterocyte-like cells expressed higher levels of *CYP2C9*, *CYP2C19,* and *CYP3A4*, compared with those of the Caco-2 cells (I). Data are expressed as the mean ± SD (n = 3; n, number of independent experiments). Relative values versus those of the adult intestine (= 1) are shown. Differences between enterocyte-like cells derived from RPChiPS771 and ChiPS18 hiPSCs were analyzed by two-way ANOVA Tukey's multiple comparisons test, significances are shown as ^∗^*P* < 0.05 or ^∗∗^*P* < 0.01.

In this study, we used the CVM in an attempt to induce the differentiation of human iPS cells into mature intestinal enterocytes. We first induced DE cells from human iPSCs on M15 cells. Day 3 DE cells were dissociated and re-plated onto CVM inserts and cultured until day 15 in a medium containing BIO and DAPT (M2), two key signals of intestinal differentiation (Ogaki et al., 2013) (Figure 1A). The immunocytochemical analysis results revealed that DE cells plated onto CVM began to actively express CDX2 on day 10 of differentiation, confirming that these cells underwent intestinal differentiation on CVM (Figure 1B).

For the maturation of iPSCs into further functional cells of the intestine, the media was switched to maturation medium (M3-0) and cultured for up to 40 days. M3-0 medium is a commercially available medium that we used for hepatic maturation (Nakai et al., 2019). At differentiation day 30, hiPS-derived intestinal cells expressed VILLIN in a polarized manner, with higher levels of expression in the apical side than in the basal side, according to confocal microscopy examination (Figure 1C).

We then analyzed the expression of intestinal markers, transporters, and metabolizing enzymes genes using real-time PCR analysis. The result revealed that a marker for crypt base columnar cells, *LGR5*, was upregulated transiently, peaking on day 5, and downregulated thereafter. The expression of an intestinal marker, *CDX2*, and an enterocyte marker, *VILLIN*, were upregulated in a mutually exclusive manner to *LGR5* expression. *CDX2* or *VILLIN* expression plateaued on days 10 and 15, respectively (Figure 1D), with their expression levels maintained at substantial levels. The expression levels of the markers are normalized to those of the adult intestine (adult intestine = 1).

Drug transporter is one of the major components to determine the intestinal absorption of drugs (Giacomini et al., 2010). Efflux transporters in the intestine, such as breast cancer resistance protein (BCRP), encoded by ATP-binding cassette family G member 2 (*ABCG2*), and P-glycoprotein (P-gp)/multidrug resistance 1 (MDR1), encoded by ATP-binding cassette family B member 1 (*ABCB1*), contribute to limiting the oral absorption of compounds by driving the efflux of substrates back into the lumen. Peptide transporter 1 (PEPT1), encoded by solute carrier family 15 member 1 (*SLC15A1*), is involved in the intestinal uptake of oligopeptides and peptide-mimetic drugs (Estudante et al., 2013). Since these transporters are important in determining the intestinal absorption of orally administered substrate drugs, we then examined their culture period-dependent expression patterns during their differentiation from hiPSCs. The mRNA expression of *ABCG2* was found to be upregulated from day 10 of differentiation, reached a plateau on day 15, whereas the *ABCB1* and *SLC15A1* mRNAs were upregulated from day 15 and reached a plateau on day 20, thereafter maintaining their expression up to day 40 (Figure 1E).

Drug metabolic enzymes also play an important role in the detoxification of xenobiotics in the enterocytes as well as hepatocytes. Of the cytochrome P450 (CYP) isoforms, CYP3A4 is a dominant metabolic enzyme in the small intestine. The mRNA expression of *CYP3A4*, *CYP2C9*, and *CYP2C19* was detected in the hiPSC-derived intestinal cells at differentiation day 20 (Figure 1F). The expression levels of *CYP2C9* and *CYP2C19* in the hiPSC-derived intestine were approximately 0.1- to 0.5-fold, while *CYP3A4* was about 0.1- to 1.3-fold of that in the adult intestine from day 30–40 of differentiation (Figure 1F). The results indicate that hiPSC-derived intestinal cells expressed intestinal markers, transporters, and CYP enzymes that resemble those of the intact human enterocytes. Therefore, we denoted these cells as hiPSC-derived enterocyte-like cells.

Then, we compared the expression levels of the markers in the hiPSC-derived enterocyte-like cells with those of the Caco-2 cells (cultured for 14 days on CVM) (Figures 1G–1I), which is the most commonly used human intestinal cell model for evaluating the intestinal absorption properties of drugs during the process of drug development. Day 3 DE cells derived from two different hiPSC lines, RPChiPS771 and ChiPS18, were cultured on CVM until differentiation day 21 and were then used for comparison with Caco-2 cells. hiPSC-derived enterocyte-like cells expressed higher levels of *VILLIN*, and lower levels of *LGR5* compared with those expressed in day 14 Caco-2 cells (Figure 1G). The expression levels of *ABCB1, ABCG2*, and *SLC15A1* were higher in the hiPSC-derived enterocyte-like cells than in day 14 Caco-2 cells (Figure 1H). RPChiPS771- and ChiPS18-derived enterocyte-like cells exhibited similar levels of *CYP3A4* expression, which was not observed in day 14 Caco-2 cells (Figure 1I).

These results indicate that CVM supports the enterocytic differentiation of hiPSC-derived DE cells, leading to the establishment of enterocyte-like cells that express mature intestinal markers, transporters, and CYP enzymes at levels higher than those expressed in Caco-2 cells.

### hiPSC-derived enterocyte-like cells exert active efflux transport and CYP3A4-mediated metabolism of drugs

**Figure 2:**
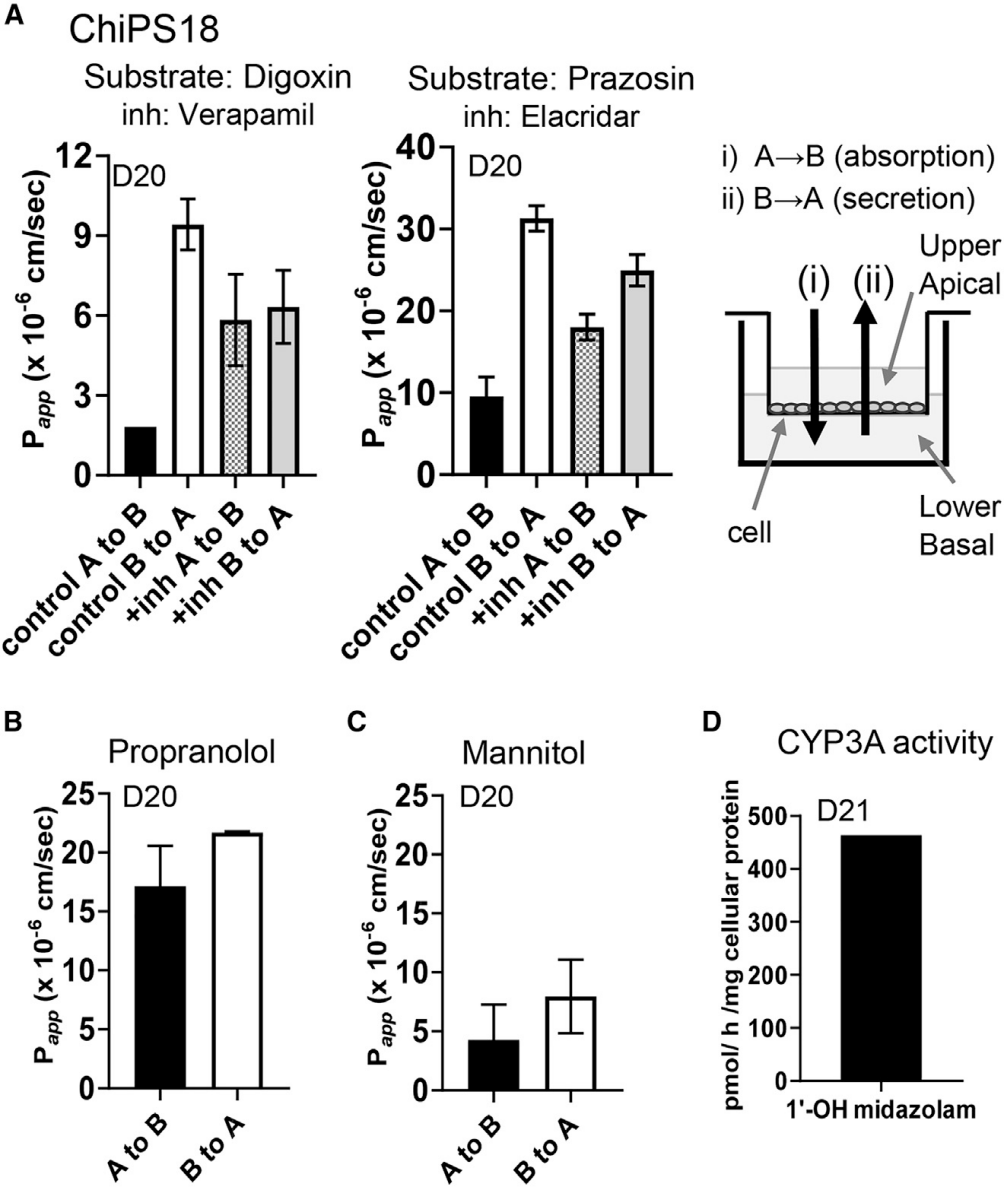
hiPSC-derived Enterocyte-Like Cells Showed the Functions of Efflux Transporters and High Permeability for Propranolol and Low Permeability of Mannitol (A) Transport activities of P-gp and BCRP were tested with their representative substrates and inhibitors. (Left) Directional transcellular transport (B-to-A and A-to-B) of digoxin (1.27 nM) (P-gp selective substrate) in the absence (control) or presence (+inh) of verapamil (P-gp inhibitor). (Middle) Directional transcellular transport (B-to-A and A-to-B) of prazosin (0.391 nM) (BCRP and P-gp substrate) in the absence (control) or presence (+inh) of elacridar (P-gp and BCRP dual inhibitor). (Right) Schematic drawing of the experiment. (B) The transport of propranolol (1.33 nM), which is mainly through the transcellular route by passive membrane permeation, was examined. (C) The transport of mannitol (1.35 nM), which is mainly through the paracellular route, was examined. (A–C) *P*_*app*_ values calculated from the slope of the time-dependent transcellular transport of substrates (Figure S1) are plotted. Data are expressed as the mean ± SD (n = 3; n, number of biological duplicates). (D) CYP3A4 activity was examined to check the formation of 1′-OH midazolam from 10 μM midazolam (n = 2). Additional experiment to confirm reproducibility of transport and metabolic enzyme CYP3A activities was performed and shown in Figure S2.

Because hiPSC-derived enterocyte-like cells were found to express *ABCG2* and *ABCB1* mRNAs at high levels, we performed a bidirectional transcellular transport assay to examine the transport activities of efflux transporters, P-gp and BCRP, in the ChiPS18-derived enterocyte-like cells on the cell culture inserts (Figure 2A, right). Basal-to-apical (B-to-A) transport exceeded apical-to-basal (A-to-B) transport of [^3^H]-digoxin, a typical substrate of P-gp (Figure 2A, left). In the presence of 100 μM verapamil, a typical inhibitor of P-gp, B-to-A transport of [^3^H]-digoxin was almost identical to A-to-B transport. Similarly, B-to-A transport of [^3^H]-prazosin, a substrate of BCRP and P-gp, exceeded its A-to-B transport. In the presence of 20 μM elacridar, a dual inhibitor of BCRP and P-gp, B-to-A transport of [^3^H]-prazosin decreased, whereas its A-to-B transport increased, confirming the partial inhibition of BCRP and P-gp by elacridar. These results suggested that the hiPSC-derived enterocytes exhibit the P-gp- and BCRP-mediated efflux of drugs (Figure 2A, Figures S1 and S2).

We then tested the transport of [^3^H]-propranolol, which is exclusively mediated by passive membrane permeation due to its high lipophilicity, and the transport of [^3^H]-mannitol, which is mediated mainly via a paracellular route due to its low molecular weight and high hydrophilicity. As a result, we could not observe directional transport of [^3^H]-propranolol and [^3^H]-mannitol across the cell monolayer (Figures 2B, 2C, and S1). The transport of [^3^H]-propranolol was much higher than that of [^3^H]-mannitol, which is reasonably explained by their different physicochemical properties.

We also tested the metabolic activity of CYP3A4 in the ChiPS18 iPSC-derived enterocyte-like cells to form 1′-OH midazolam from midazolam, which is known to be selectively mediated by CYP3A4 (Andrew Williams et al., 2002). The formation of 1′-OH midazolam could be detected by liquid chromatography-tandem mass spectrometry (LC-MS/MS) at 463.8 pmol/h/mg cellular protein (Figure 2D). Additional experiment was performed to confirm the metabolic enzyme activity of CYP3A by detecting the formation of 6β-OH testosterone from testosterone by LC-MS/MS (Figure S2). Taken together, our results indicate that the iPSC-derived enterocyte-like cells have efflux transporter activities of both P-gp and BCRP, and CYP3A4-mediated metabolic activity.

### Investigation of the maturation procedure to generate hiPSC-derived matured enterocytes

**Figure 3:**
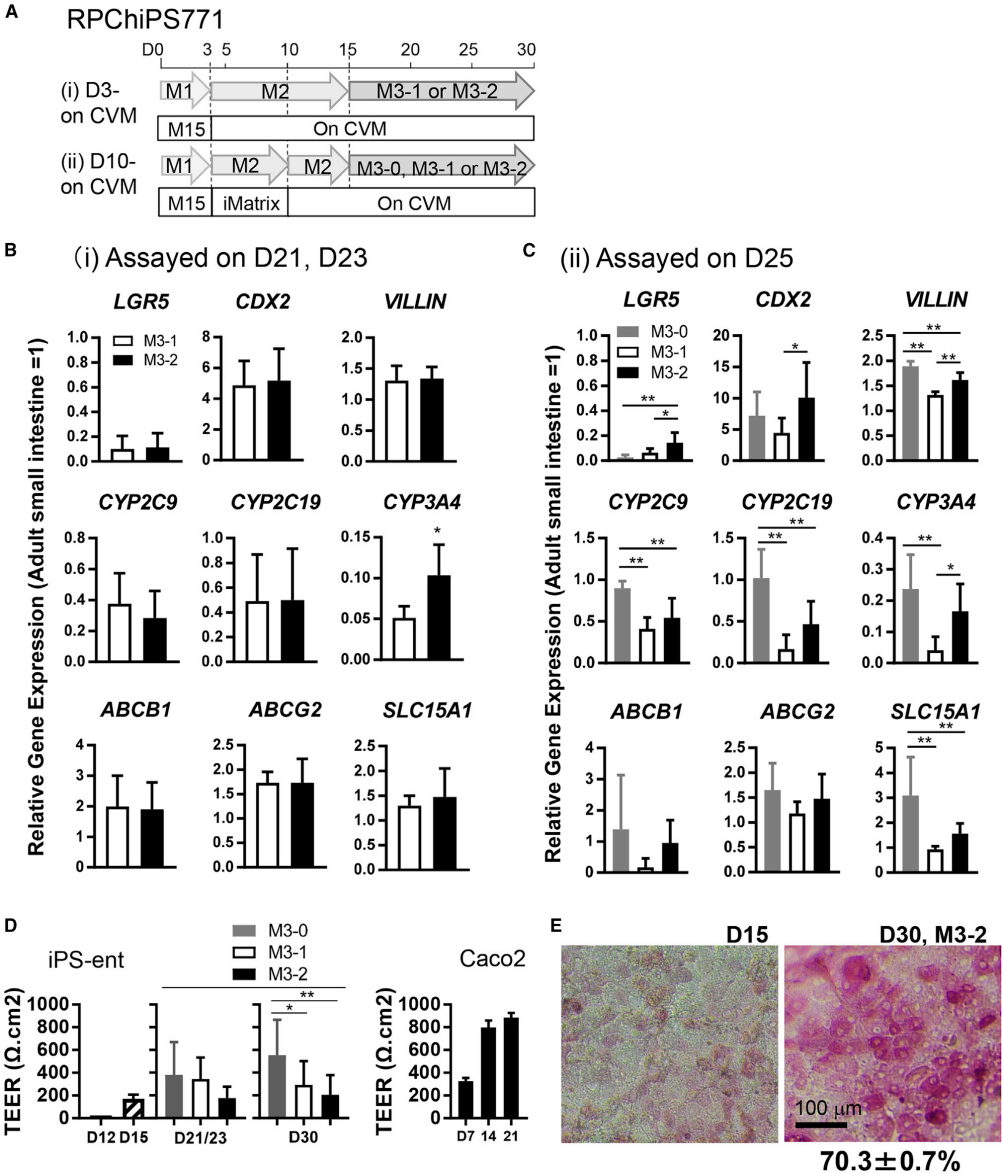
Alternative Maturation Procedure to Generate hiPS-derived Enterocyte-Like Cells (A) A schematic drawing of the experimental design. (B and C) The expression of differentiation markers, transporters, and metabolizing enzymes in RPChiPS771-derived enterocyte-like cells cultured under protocol (i) assayed on day 21 or 23 (D21, D23) (B) or protocol (ii) assayed on day 25 (D25) (C) are shown. (D) TEER values of iPS-derived enterocyte-like cells cultured under protocol (ii) (left) or Caco-2 cells (right). (E) The activity staining of ALP was performed with RPChiPS771-derived enterocyte-like cells on D15 and D30. Data are expressed as the mean ± SD (n = 3; n, independent experiments). Relative values versus those of the adult intestine (=1) are shown (B, C). Differences between groups were analyzed by Student’s t test (B), or one-way ANOVA Tukey's multiple comparisons test (C, D), ^∗^p < 0.05, ^∗∗^p < 0.01. Scale bar, 100 μm.

Our results indicate that functional enterocyte-like cells are derived by culturing DE cells on CVM inserts and differentiated into the intestinal lineages in media containing BIO and DAPT during days 3–15, before maturing by culturing in maturation medium from days 15–30 (Figures 1A and 3A). Initially, we used a commercially available maturation medium (M3-0) (Figure 1A). We then tested the components for their maturation into enterocyte-like cells and focused on the comparison between using M3-1 or M3-2 medium during days 15–30 (Figure 3A, protocol i). We also tested a two-step procedure by plating day 3 DE onto iMatrix precoated plates, then replating them on CVM on day 10 and culturing until day 30, and compared three maturation medium, M3-0, M3-1 and M3-2, for the maturation step during days 15–30 (Figure 3A, protocol ii) (Figure 3A).

In this experiment, we used RPChiPS771 cells, since RPChiPS771-derived enterocyte-like cells exhibit transporter expression levels more similar to those of the adult intestine than hiPSC18 cells (Figure 1H) and might represent its physiological characteristics. Gene expression analyses revealed that iPSC-derived intestinal cells cultured in M3-1 or M3-2 (protocol i) both expressed high levels of *CDX2* or *VILLIN* on day 21 (Figure 3B) and downregulated the expression of *LGR5*. *CYP* metabolizing enzymes and transporters are also expressed, suggesting that these cells differentiated into matured enterocyte-like cells under both conditions. Cells cultured in the M3-2 medium showed significantly higher levels of *CYP3A4* expression compared with cells cultured in the M3-1 medium (Figure 3B).

We then compared among three maturation media, M3-0, M3-1, and M3-2 (protocol ii). RPChiPS771-derived enterocyte-like cells cultured in M3-0 gave the highest expression levels of *CDX2*, *VILLIN*, *CYP* metabolizing enzymes and transporters, assayed on day 25 (D25). Compared with those in M3-1, the derived enterocyte-like cells cultured in M3-2 showed a significantly higher level of markers such as *VILLIN* and *CYP3A4* (Figure 3C).

As the maturation of intestinal epithelial cells is characterized by the formation of a rigid cell monolayer that functions as a barrier, we assayed the integrity of the cell layer by measuring the transepithelial electrical resistance (TEER) values of the RPChiPS771-derived enterocyte-like cells and Caco-2 cells. RPChiPS771-derived cells cultured in M3-0, M3-1, or M3-2 medium reached a plateau at 554, 292, or 204 Ω·cm^2^ on day 30, respectively. TEER value in RPChiPS771-derived enterocyte-like cells cultured in M3-0 was significantly higher than that cultured in the M3-1 or M3-2 medium (Figure 3D). Caco-2 cells grown on CVM inserts showed a high TEER value, at approximately 885 Ω·cm^2^ on day 21 (Figure 3D). Caco-2 cells are reported to develop an unphysiologically tight junction (Artursson et al., 1993; Gupta et al., 2013). The results suggest that iPSC-derived enterocyte-like cells exhibit the integrity of cell monolayer on the culture insert with a more physiologic TEER value than that of Caco-2 cells.

We then adopted protocol ii and used M3-2 for maturation medium for subsequent experiments. We also tested another hiPSC line, ChiPS12, and found that the derived enterocyte-like cells expressed transporters and CYP metabolizing enzymes (Figure S3A). We then used ChiPS18 cells and tested if Matrigel also could be used for supporting intestinal differentiation. We found that ChiPS18-derived enterocyte-like cells grown on Matrigel expressed transporter and CYP metabolizing enzymes, although the *ABCG2* expression levels were lower, *CYP2C9* and *CYP2C19* expression levels were higher than those grown on CVM (Figure S3B). Under our protocol, the activity of alkaline phosphatase (ALP) that marks mature enterocytes (Sato et al., 2011) was observed in 70.3% ± 0.7% of RPChiPS771-derived enterocyte-like cells on D30, thereby suggesting the heterogeneous characteristics of the induced enterocytes (Figure 3E), which might consist of immature enterocytes and other mature cell types of the intestine.

### Components for promoting maturation into hiPS-derived enterocyte-like cells

**Figure 4:**
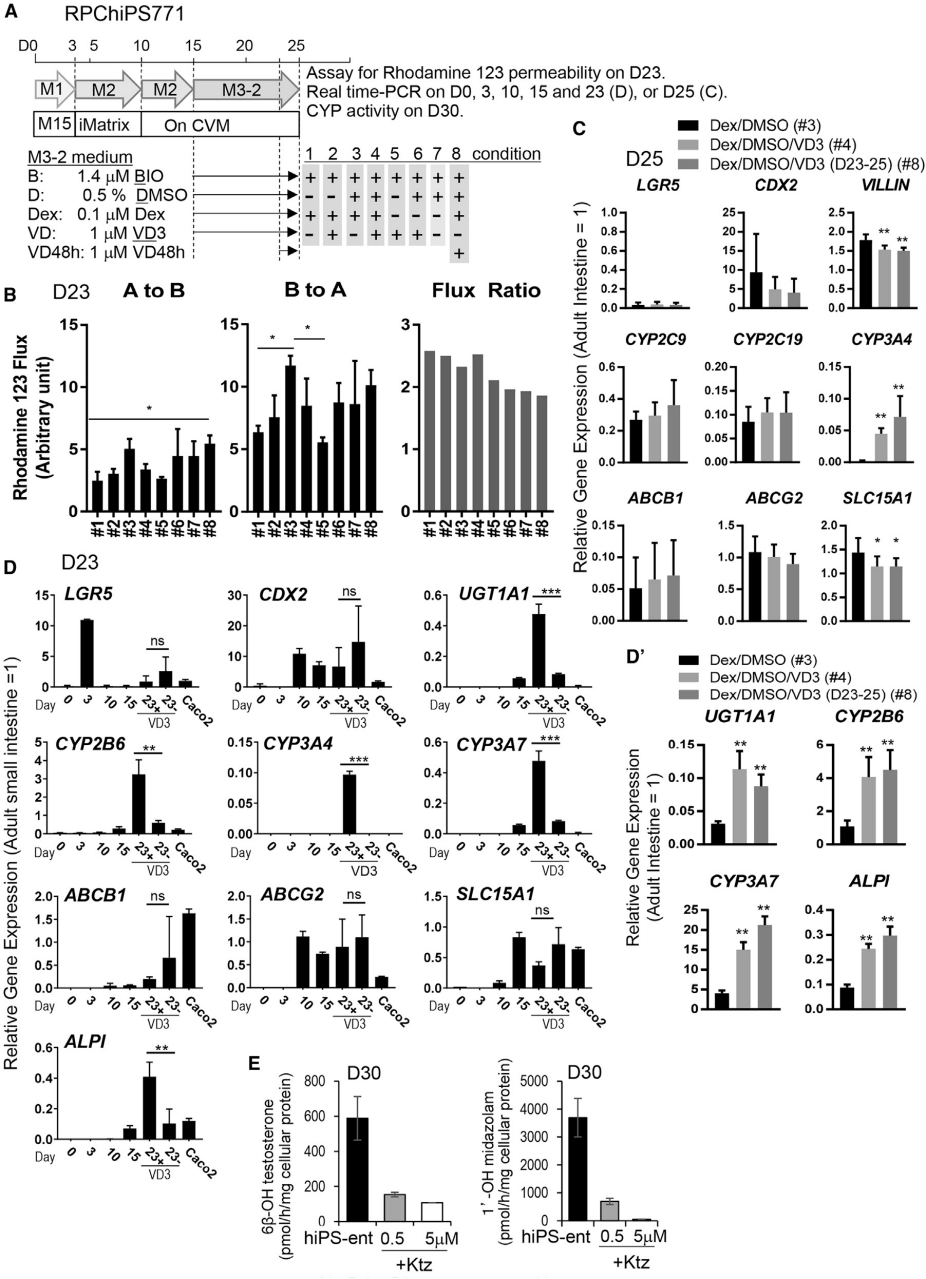
Components for Maturation into hiPSC-derived Enterocyte-Like Cells (A) A schematic drawing of the experimental procedure for differentiating hiPS RPChiPS771 into enterocytes. Maturation was performed by culturing the cells in M3-2, under eight conditions, either with a full set of factors or in the absence of 1 or 2 certain factors, as noted. Differentiated cells were assayed for rhodamine 123 permeability (B), real-time PCR analysis (C, D), and CYP3A activity (E). (B) Directional transcellular transport of fluorescence derived from rhodamine 123 (left: A to B; middle: B to A) or the flux ratio (right) are shown. (C) Expression levels of the gene markers, on maturation under three different conditions (condition #3, 4, 8). (D) Time-dependent expressions of intestinal marker genes (*LGR5* and *CDX2*), metabolic enzymes (*UGT1A1*, *CYP2B6*, *CYP3A4,* and *CYP3A7*), transporters (*ABCB1*, *ABCG2,* and *SLC15A1*), and a mature marker *ALPI*, during differentiation and with or without VD3. (D′) Additional experiment was performed to confirm the reproducibility of upregulation of UGT1A1, CYP2V6, CYP3A7, and ALPI expression by VD3. (E) CYP3A4 activity was examined, using testosterone (left) or midazolam (right) as substrates, and quantification of their metabolites was performed by LC-MS/MS. Ktz: ketoconazole (CYP3A inhibitor). Data are expressed as the mean ± SD (n = 3–4; n, independent experiments). (C, D, D′) Relative values versus those of the adult intestine (= 1) are shown. Differences versus controls or between groups were analyzed by one-way ANOVA Tukey's multiple comparisons test (B, C, D′), or Student’s t test between VD3 ± (D); ^∗^p < 0.05, ^∗∗^p < 0.01, or ^∗∗∗^p < 0.001. ns, not significant.

We then examined the effect of each component in the maturation medium M3-2 in detail. We performed differentiation using M3-2 as the maturation medium, with the absence of one or two components throughout the maturation period (day 15–25), and also tested the addition of 1α,25-dihydroxyvitamin D3 (VD3) for the last 48 h of maturation (day 23–25) (Figure 4A). We tested a total of eight conditions for the maturation of the hiPSC-derived intestinal progenitor cells into functional enterocyte-like cells. Then, we evaluated transporter activity of P-gp in the hiPSC-derived cells on day 25 by measuring the directional (A-to-B and B-to-A) transport of rhodamine123, a typical P-gp substrate, using iPSC-derived enterocyte-like epitheliums to investigate the effect of each compound in the medium on the flux ratio ([B-to-A transport]/[A-to-B transport]) of rhodamine 123. A significantly higher B-to-A transport was observed in condition #3, in comparison with condition #1 and #5 (Figure 4B, middle). A-to-B transport was not significantly different between these conditions (Figure 4B, left). We, therefore, concluded that DMSO and dexamethasone (Dex) might be important in the maturation of enterocyte-like cells for determining transporter activity. In the absence of Dex (condition #6, 7) or without Dex and DMSO (condition #5), a low rhodamine 123 flux ratio was observed (Figure 4B, right). The addition of VD3 for the last 48 h (condition #8) also yielded intestinal cells with a low (<2) rhodamine 123 flux ratio (Figure 4B, right). However, the presence of VD3 during the past 48 h was essential for the expression of the metabolic enzyme *CYP3A4* gene (Figure 4C). Therefore, we adopted the M3-2 medium containing BIO, DMSO, Dex, and VD3 for the functional maturation of hiPSC-derived intestinal cells.

We then used M3-2 for maturation and evaluated the time-dependent expression of marker genes (Figure 4D). We found that under the M3-2 maturation conditions, the expression of the stem cell marker gene *LGR5* decreased rapidly after day 3. The expression levels of UDP glucuronosyltransferase 1-1 (*UGT1A1*) and various isoforms of P450 metabolizing enzymes (*CYP2B6*, *CYP3A4*, and *CYP3A7*) increased in the presence of VD3 compared with the controls without VD3. The expression levels of uptake transporter, *SLC15A1*, and efflux transporters, *ABCB1* and *ABCG2*, were not affected by VD3. The expression level of the mature enterocyte marker Alkaline Phosphatase, Intestinal (*ALPI*) (Sato et al., 2011) was upregulated in the presence of VD3 (Figures 4D and 4D′).

Using this M3-2 medium, we then tested for the metabolic activity of CYP3A by measuring the rate of hydroxylation reaction of its typical probe substrates, testosterone and midazolam, to form 6β-OH testosterone and 1′-OH midazolam, respectively. hiPSC-derived enterocyte-like cells on day 30 exhibited metabolizing activity, and metabolites produced by CYP3A were at high levels of approximately 0.6 nmol/h/mg cellular protein for 6β-OH testosterone or 3.7 nmol/h/mg cellular protein for 1′-OH midazolam, respectively (Figure 4E). Moreover, ketoconazole, a potent CYP3A inhibitor, suppressed the metabolite formation of testosterone and midazolam, further supporting that these metabolites are produced by CYP3A.

Given the above results, culturing the hiPSC-derived intestinal progenitor cells on CVM with a medium containing DMSO, BIO, Dex, and VD3 gave rise to mature enterocyte-like cells exhibiting high integrity of cell monolayer, transport activities of efflux transporters, and metabolic activity of CYP3A4, thereby resembling adult enterocytes.

### hiPSC enterocyte-like cells as a model for the prediction of apparent drug absorption

**Figure 5:**
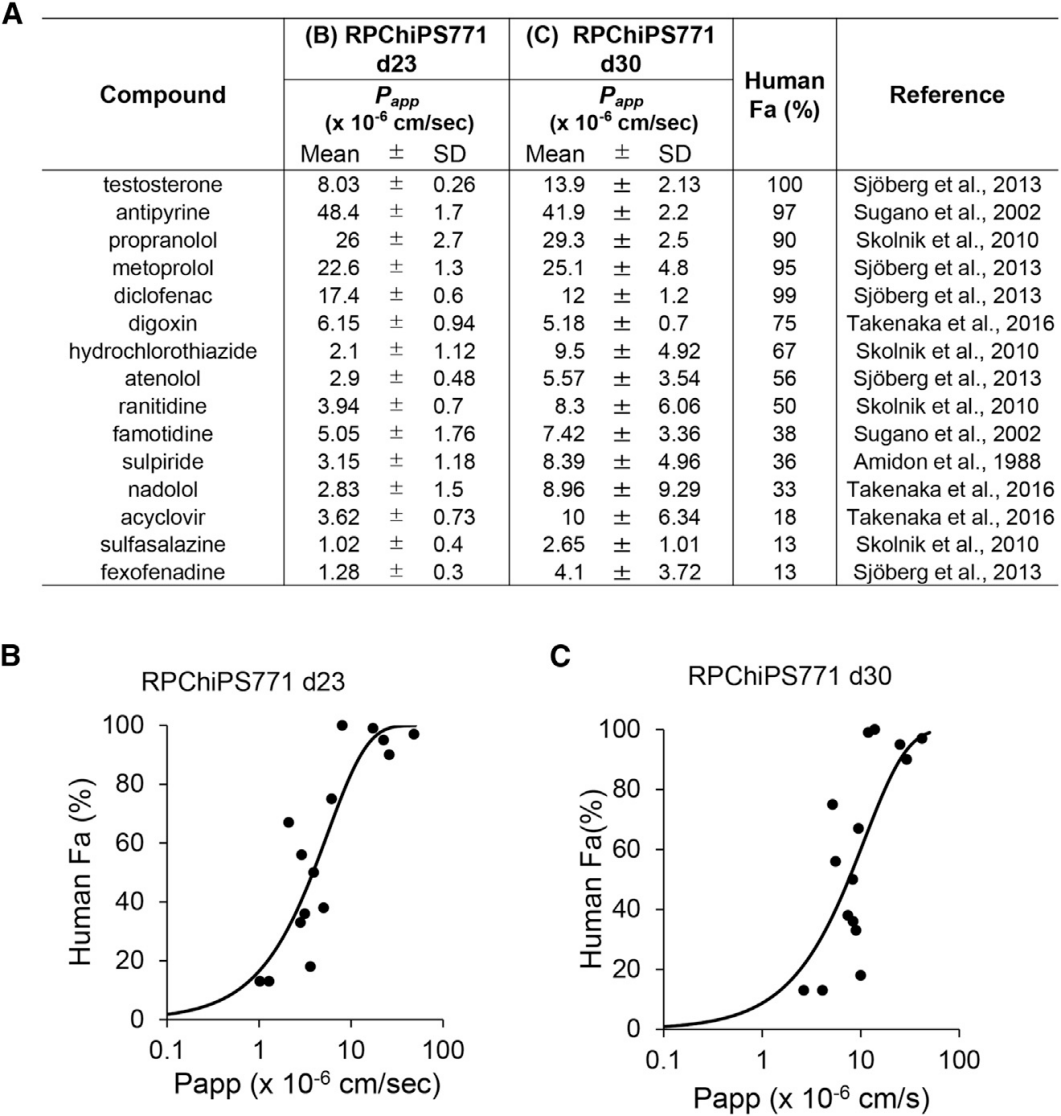
A Good Correlation of *in vitro P*_*app*_ in hiPSC-derived Enterocyte-like Cells with Fa Values of Test Drugs in Humans (A) Human RPChiPS771 iPSC-derived enterocytes matured using M3-2 medium were used for evaluating the apparent permeability of 15 test drugs on day 23 (B) or D30 (C) of differentiation. Drugs with known Fa values and the references used in this study are shown in the list. Data are expressed as the mean ± SD (n = 4; n, number of duplicates) (B and C). The mean values of *P*_*app*_ against Fa of the drugs are plotted, which showed a good correlation and sigmoidal relationship with the coefficient of determination *R*^2^ = 0.749 (B), or *R*^2^ = 0.553 (C).

We then examined if our present hiPSC-derived enterocyte-like cells would be useful for predicting *in vivo* intestinal absorption. We performed a permeability test using the RPChiPS771 iPS-derived enterocyte-like cells with 15 compounds to investigate whether the apparent permeability coefficients (*P*_*app*_) correlated with the fraction of oral dose absorbed from the intestinal lumen (Fa) of the compounds in humans (Figure 5A) (Amidon et al., 1988; Sjöberg et al., 2013; Skolnik et al., 2010; Sugano et al., 2002; Takenaka et al., 2016; Tavelin et al., 2003; Xiao et al., 2019). The compounds tested included highly permeable compounds, such as testosterone, antipyrine, propranolol, metoprolol, and diclofenac; moderately permeable compounds, such as hydrochlorothiazide, atenolol, sulpiride, and nadolol; P-gp substrates, such as digoxin, famotidine, ranitidine, and fexofenadine; and BCRP substrate, such as sulfasalazine. The *P*_*app*_ of highly permeable compounds were 8.03‒48.4 × 10^−6^ cm/s, whereas the *P*_app_ of moderately permeable compounds were 2.10‒3.15 × 10^−6^ cm/s (Figure 5B). The sigmoidal correlation between the corresponding Fa values and *P*_*app*_ in hiPSC-derived enterocyte-like cell monolayer revealed a coefficient of determination of *R*^2^ = 0.749 (Figure 5B). We performed additional experiment on d30 RPChiPS771-derived enterocyte-like cells (Figure 5C) and obtained a coefficient of determination of *R*^2^ = 0.553 (Figure 5C). These results suggest that the hiPS-derived enterocyte-like cells may serve as an appropriate *in vitro* model for predicting the intestinal absorption of drug candidates in the drug development.

## Discussion

In this study, we established an efficient culture procedure for generating enterocyte-like cells from hiPSCs by culturing the hiPSC-derived endoderm or intestine progenitor cells on CVM. We found that CVM is a good substrate for the induction and maintenance of mature enterocyte cells. We previously reported that we could generate CDX2-positive intestine cells by culturing hiPSCs on M15 cells and the addition of BIO, a Wnt signal activator, and DAPT, a Notch signal inhibitor. The hiPSC-derived CDX2-positive intestine progenitor cells cultured on CVM differentiated to form enterocyte-like cells. Using any of the three different maturation conditions, we demonstrated the successful generation of hiPSC-derived enterocyte-like cells with high levels of mRNA expression for efflux transporters *ABCB1* and *ABCG2* and the uptake transporter *SLC15A1*, with expression maintained at substantial levels from differentiation day 21 up to at least day 30 (Figures 1 and 3). The derived enterocyte-like cells also showed higher *CYP3A4* mRNA expression (Figures 1, 3, and 4) than those of the day 14 Caco-2 cells (Figure 1). Using three hiPSC lines, ChiPS18, RPChiPS771, and ChiPS12 cells, we found that enterocyte-like cells derived from either cell line exhibit similar mRNA expression levels of *ABCB1* and various CYP isoforms. Consistent with mRNA expression profiles, we were able to detect a directional transport of P-gp and/or BCRP substrates, digoxin, prazosin, and rhodamine 123, and CYP3A-mediated metabolism of midazolam and testosterone during this period (Figures 2 and 4).

It is reported that a switch occurs from CYP3A7 as a predominant isoform in the fetal to CYP3A4 in the adult liver during development (Stevens et al., 2003). The expression of CYP2C9 and CYP2C19 expression levels in children or adults are higher than those expressed in the fetal liver (Zane et al., 2018). We, therefore, considered that expression of higher CYP metabolizing enzymes to be of higher maturity, and tried to find conditions that gave higher CYP enzyme expression levels. The examination of the medium for the maturation of intestine progenitor cells into functional enterocytes revealed that the addition of activated VD3 and Dex is beneficial for the efflux activity of P-gp and that activated VD3 induced the expression of drug-metabolizing enzymes, such as *CYP3A4*, *UGT1A1*, and *CYP2B6* (Figure 4). It is reported that Dex upregulates the expression of P-gp in human cultured liver cell line and retinal pigment epithelium, which might contribute to the elevated efflux ratio of substrates (Zhang et al., 2012; Zhao et al., 2015). VD3 has been reported to induce the expression of *CYP3A4*, as well as the three key D3-hydroxylase gene transcripts (25-hydroxylase, *CYP2*7A; 24-hydroxylase, *CYP24*; *1,* α-hydroxylase, *CYP27B1*) in human fetal small intestine cells aged 15 to 20 weeks (Theodoropoulos et al., 2003). The VD receptor was reported to be expressed in the human small intestine of fetuses (Delvin et al., 1996). These reports suggest the role of the auto/paracrine action of VD3 in the regulation of human gut development.

With regard to the directional transport of digoxin and prazosin (Figure 2, using M3-0), or rhodamine 123 (Figure 4, using M3-2), the flux ratios obtained here suggest that the transport activities of efflux transporters were comparable to the results previously reported in Caco-2 cells (Djuv and Nilsen, 2008; Takenaka et al., 2014; Wright et al., 2011) or to those obtained in the human small intestine (Sjöberg et al., 2013; Speer et al., 2019; Takenaka et al., 2014).

Caco-2 cells are characterized by limited paracellular transport compared with intact intestinal epithelial cells due to the more rigid, tight junction of Caco-2 cells (Takenaka et al., 2014). hiPSC-derived enterocytes show a much lower TEER value compared with that of Caco-2 cells (Figure 3D), suggesting higher levels of paracellular transport in hiPSC-derived enterocyte-like cells than in Caco-2 cells. On the other hand, the passive permeability of propranolol was 41.9 × 10^−6^ cm/s in the Caco-2 cells (Artursson, 1990), which is comparable to our present result. To determine whether hiPSC-derived enterocyte-like cells can withstand practical use in drug development, the correlation of the permeabilities of 15 drugs with the corresponding Fa values was examined. *P*_*app*_ values spanning from 1.0 × 10^−6^ cm/s to 48 × 10^−6^ cm/s were found to be correlated to the reported Fa values (Figure 5). The sigmoidal correlation between the *P*_app_ of 15 test compounds with diverse human Fa values using primary human small intestinal cells and the corresponding human Fa value was 0.779 (Takenaka et al., 2014). Moreover, because test compounds include substrates of efflux transporters (P-gp, BCRP), such a good correlation for all tested compounds implies that transporter functions are also maintained in our hiPSC-derived enterocyte-like cells at similar levels in intact human intestine. Our results, therefore, support the fact that hiPSC-derived enterocytes show characteristics that resemble those of the human adult intestine, and may thus be useful for the prediction of intestinal absorption by gut epithelial cells. We also performed a permeability test with the hiPSC-derived enterocytes using the maturation medium M3-0 with the same test drugs and obtained a similar coefficient of determination *R*^2^ = 0.775 (SY, unpublished data).

The expression levels of the CYP isoforms are shown as the expression level relative to that of human adult intestinal cells. In the hiPSC-derived enterocyte-like cells established in this study, the mRNA expression of *CYP2C9* and *CYP2C19* was also expressed at a level approximately 0.1- to 1.5-fold that observed in the human intestinal cells, while the mRNA expression level of *CYP3A4* was approximately 0.05-fold (under condition M3-0; Figure 1) or 0.1-fold (under condition M3-2; Figure 3) of that in human intestinal cells (Figures 1I and 3). On the other hand, 1′-OH midazolam formation by iPSC-derived enterocyte-like cells generated under condition M3-2 assayed on D30 (Figure 4) was approximately 8-fold of that obtained under condition M3-0 assayed on D20 (Figure 2). Considering a 2-fold initial midazolam concentration difference, CYP3A enzyme activity observed in Figure 4E was approximately 4-fold higher than that in Figure 2D. However, because we observed that the metabolite amount reached a plateau at 30 min (SY unpublished), the metabolite rate shown in Figure 2D might be underestimated.

hiPSC-derived enterocyte-like cells show a 15-fold higher expression of *CYP3A7* compared with the adult intestine, which might be neglectable since CYP3A7 is a major isoform expressed in the fetal, but not in the adult intestine (Figure 4). Also, CYP3A4 is reported to catalyze the formation of 1′-OH midazolam *in vitro* at approximately >600-fold compared to CYP3A7 (Andrew Williams et al., 2002). Therefore, the observed metabolic activity of midazolam clearance is considered to be originated mainly from CYP3A4. The activities of other drug-metabolizing enzymes need to be measured in future studies using their specific substrates. Our above results extend our previous report that DE cells treated with BIO and DAPT differentiated into intestine progenitor cells, which can be further directed into enterocyte-like cells by culturing under either maturation media M3-0, M3-1, or M3-2.

For the preparation of hiPSC-derived enterocyte-like cells, we performed endoderm differentiation into M15 cells, intestinal progenitor cell differentiation on iMatrix, and the maturation of cells into enterocyte-like cells on CVM in a stepwise manner. The iPS-derived endoderm and intestinal progenitor cells were then cryopreserved. Upon freeze-thaw and plating onto CVM, the cells re-adopted differentiation and could be readily used for the generation of enterocyte-like cells. We routinely started from one 100 mm dish of undifferentiated iPS cells (5 × 10^5^ cells) to obtain approximately 1.5–2 × 10^7^ endoderm or intestinal progenitor cells, which finally gave rise to iPS-derived enterocyte-like cells equivalent to approximately 90-125 culture inserts (for 24 multi-well plates). By increasing the number of undifferentiated hiPSCs, the large-scale preparation of enterocyte-like cells is feasible. We established a simple and reproducible differentiation method suitable for the evaluation of the intestinal absorption of drugs in humans.

In conclusion, we succeeded in generating functional enterocytes that exhibit directional transport activities driven by efflux transporters, the metabolic activity mediated by CYP3A4 and that can be used as an *in vitro* model for the prediction of human Fa values of drugs. Our results indicate that the hiPSC-derived enterocyte-like cells established in this study could be used for the quantitative prediction of intestinal absorption of drugs in humans under special occasions such as alteration of the functions of transporters/metabolic enzymes by drug-drug interactions as well as normal condition.

## Experimental procedures

### Human iPS cell lines

Two human iPS cell lines, ChiPS18 (Asplund et al., 2016) (Takara Bio, Kusatsu, Japan), RPChiPS771 cells (ReproCell, Yokohama, Japan), or ChiPS12 cells (Takara Bio) were used. Undifferentiated iPS cells were maintained in AK02N StemFit media (Ajinomoto, Tokyo, Japan) on cell culture dishes precoated with Synthemax II (Corning, Corning, NY, USA). For methionine deprivation, ChiPS18, ChiPS12, and RPChiPS771 cells were cultured in the Methionine-deprived KA01 medium (Ajinomoto).

### Differentiation of iPS cells into intestinal cells

To initiate differentiation, undifferentiated ChiPS12, ChiPS18, or RPChiPS771 cells were first differentiated into the DE on M15 feeder cells and cultured in the differentiation medium M1, then dissociated and either plated directly for further differentiation or cryopreserved. For intestine differentiation, D3 DE cells were plated onto rehydrated vitrigel (CV) membrane 24-well inserts (ad-MED Vitrigel 2, Kanto Chemical Co., Inc., Tokyo, Japan, culture area: 0.33 cm^2^/insert), and cultured in M2 for day (D) 4-D15, then changed to M3 (M3-0, M3-1 or M3-2) for D15-D21, or up to D40. Detailed information is outlined in the Supplemental Experimental Procedures.

### The human adult small intestine

Total RNA of the human adult small intestine (ASI) (Takara Bio, 63653); Lot No. 1012049A was used in Figures 1 and 3B; Lot. No. 1901903A was used in Figures 1, 3C, 4, and S3. No significant differences were observed between the two lots of human ASI. The levels of gene expressions are shown as fold against ASI (ASI = 1). Normal human adult small intestines were pooled from five male/female Caucasians ages ranged from 20 to 61.

### Transcellular transport assays and measurement of CYP3A metabolite

In Figures 2 and S1, for assessing the transporter activity of P-gp or BCRP, time-dependent directional transport of [^3^H]-digoxin or [^3^H]-prazosin, respectively, was measured in the absence or presence of their specific inhibitors. For assessing transcellular transport and paracellular transport across cell monolayer, [^3^H]-propranolol and [^3^H]-mannitol were tested, respectively. Details are outlined in the Supplemental Experimental Procedures.

### Permeability measurements

The apparent permeability coefficient (*P*_*app*_) for each of the 15 compounds was determined by incubating the hiPS-derived enterocyte-like cells with buffer containing substrate ± inhibitor at 37°C for 2 h, outlined in the Supplemental Experimental Procedures. The unlabeled compounds were analyzed by LC-MS/MS. The detailed conditions for the analyses of the compounds are shown in Table S1.

### Rhodamine 123 permeability assay by fluorescence detection

Rhodamine 123 (10 μM; Dojindo, Kumamoto, Japan, R233) was used as a substrate to assess the transporter activity of P-gp. The flux of rhodamine 123 was determined by a Luminometer (GloMax Microplate Luminometer, Promega). The flux ratio of rhodamine 123 was calculated as follows. Flux ratio = *P*_*app*, basolateral to apical_/*P*_*app*, apical to basolateral_

### Measurement for CYP metabolites

In Figure 4, the measurement for CYP metabolites was done by replacing the hiPSC-derived enterocyte culture medium (M3-2) with transport buffer containing substrates (midazolam 20 μM or testosterone 50 μM) and incubated at 37°C for 120 min, with or without a potent CYP3A4 inhibitor, ketoconazole at 0.5 or 5 μM, outlined in the Supplemental Experimental Procedures. Detailed LC-MS/MS analysis conditions of the metabolite, 1′-OH midazolam or 6β-OH testosterone, respectively, are listed in Table S1.

### Statistics

Data are expressed as the mean ± SD. Differences between groups were analyzed by Student’s t tests or ANOVA multiple comparisons tests. The respective statistical analysis and p values are noted in each figure legend. ^∗^p < 0.05, ^∗∗^p < 0.01, or ^∗∗∗^p < 0.001, are considered to be significant.

## Author contributions

S.Y. and N.S. designed the experiments, and acquired, analyzed, and interpreted data. T.H., K.I., R.I., and S.L. designed the experiments, and acquired and analyzed the data. T.S. acquired and analyzed the data. T.W. and M.I. took part in the generation and analysis of CVM. K.M. and H.K. designed, acquired, and analyzed a part of the experiments, and discussed the data. S.K. provided conceptual input, discussion, writing, and revision of the manuscript, approved the final version of the manuscript, and obtained funding.

## Conflicts of interest

A part of the research was conducted with a research fund from Shionogi & Co., Ltd.

T.W., M.I., K.M., H.K., N.S., and S.K. are inventors on related patent applications.

## Supplemental Information

Document S1. Supplemental experimental procedures, Figures S1–S3, and Tables S1 and S2

Document S2. Article plus supplemental information
