# Supplementary material for: Generation of Human-Induced Pluripotent Stem Cell-Derived Functional Enterocyte-Like Cells for Pharmacokinetic Studies
Source: Stem Cell Reports. 2021 Jan 28;16(2):295–308. doi: 10.1016/j.stemcr.2020.12.017 (PMC7878837; doi:10.1016/j.stemcr.2020.12.017)
Supplement: Document S1. Supplemental experimental procedures, Figures S1–S3, and Tables S1 and S2 [file mmc1.pdf]

Stem Cell Reports, Volume *16*

Supplemental Information

Generation of Human-Induced Pluripotent Stem Cell-Derived Functional Enterocyte-Like Cells for Pharmacokinetic Studies

Shinpei Yoshida, Takayuki Honjo, Keita Iino, Ryunosuke Ishibe, Sylvia Leo, Tomoka Shimada, Teruhiko Watanabe, Masaya Ishikawa, Kazuya Maeda, Hiroyuki Kusuhara, Nobuaki Shiraki, and Shoen Kume

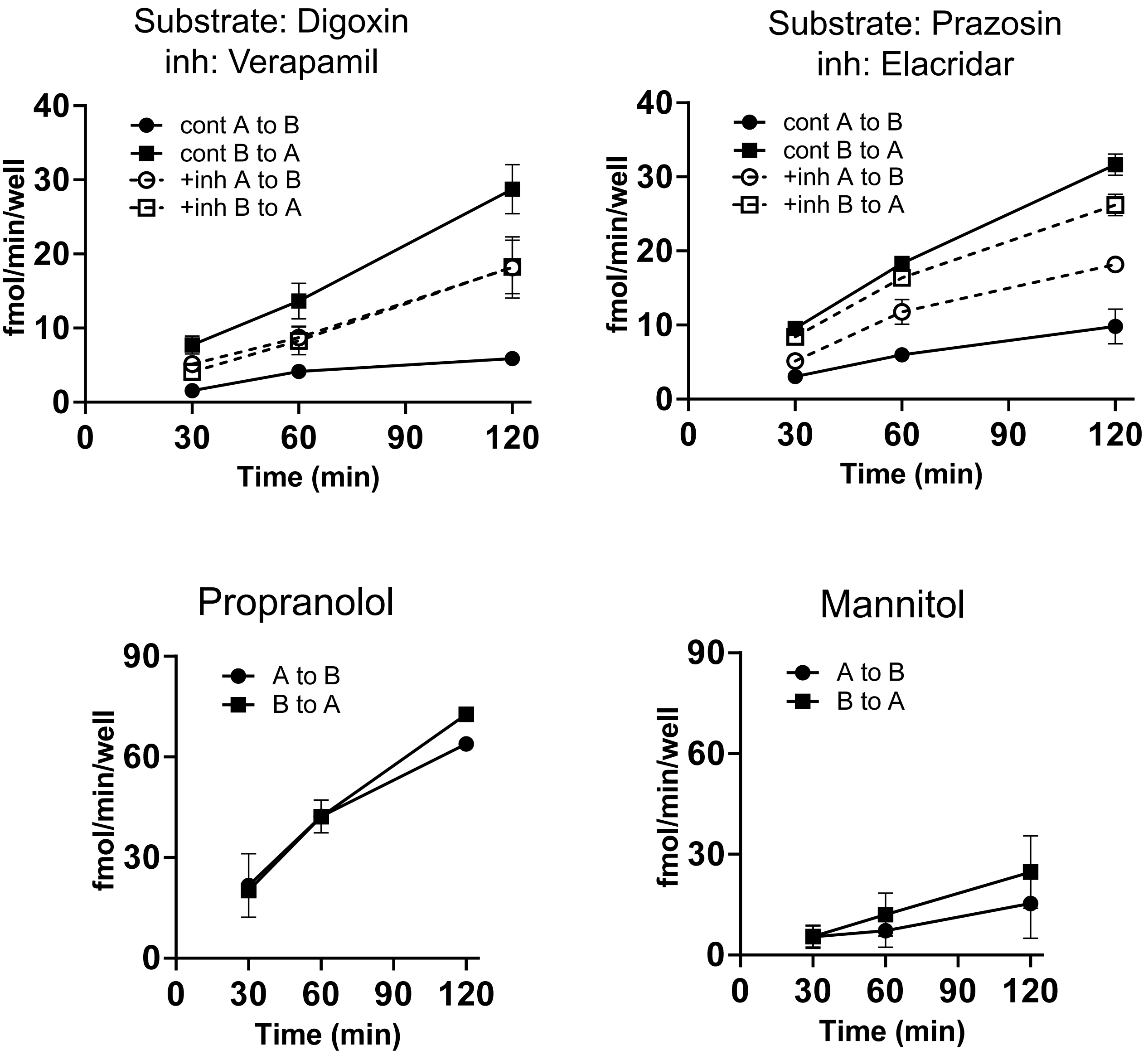

Figure S1 Time-dependent apical-to-basal and basal-to-apical transport of digoxin, prazosin, propranolol and mannitol across the monolayer of hiPSC-derived enterocyte like cells. Related to Figure 2.

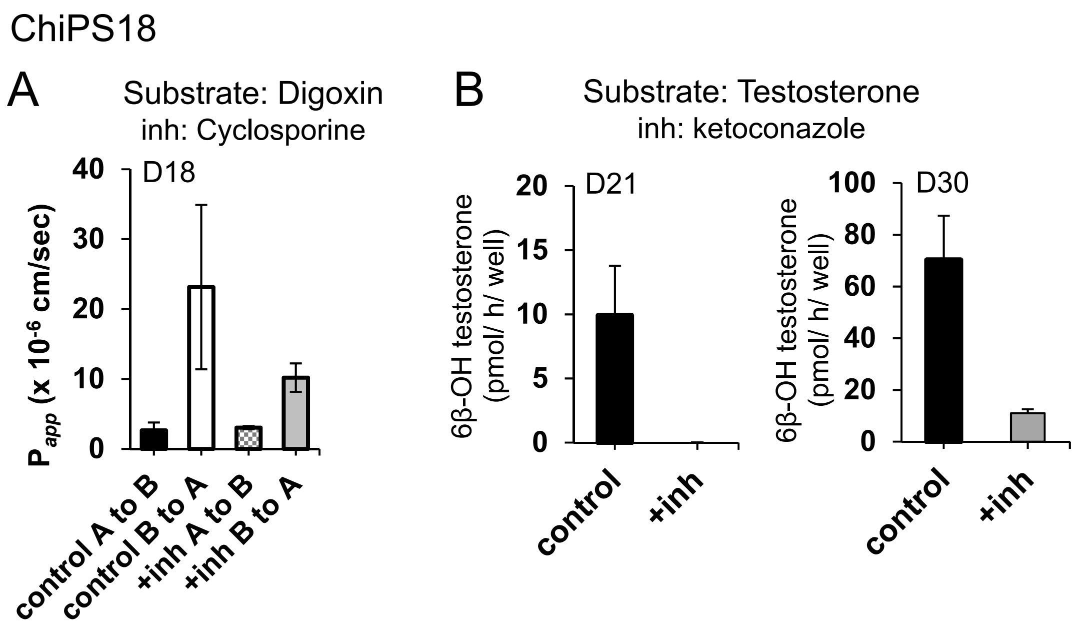

Figure S2 hiPSC-derived enterocyte-like cells showed the functions of efflux transporters and metabolic enzyme activities. Related to Figure 2. Additional independent experiments using ChiPS18 human iPSCs were performed to confirm (A) the transport and (B) CYP3A metabolic enzyme activities of the derived intestinal enterocyte-like cells. Related to Figure 2. Data are shown as the mean ± SD (n=3; n, number of replicates. Measurements were performed by analyzing the unlabeled compounds by LC-MS/MS.

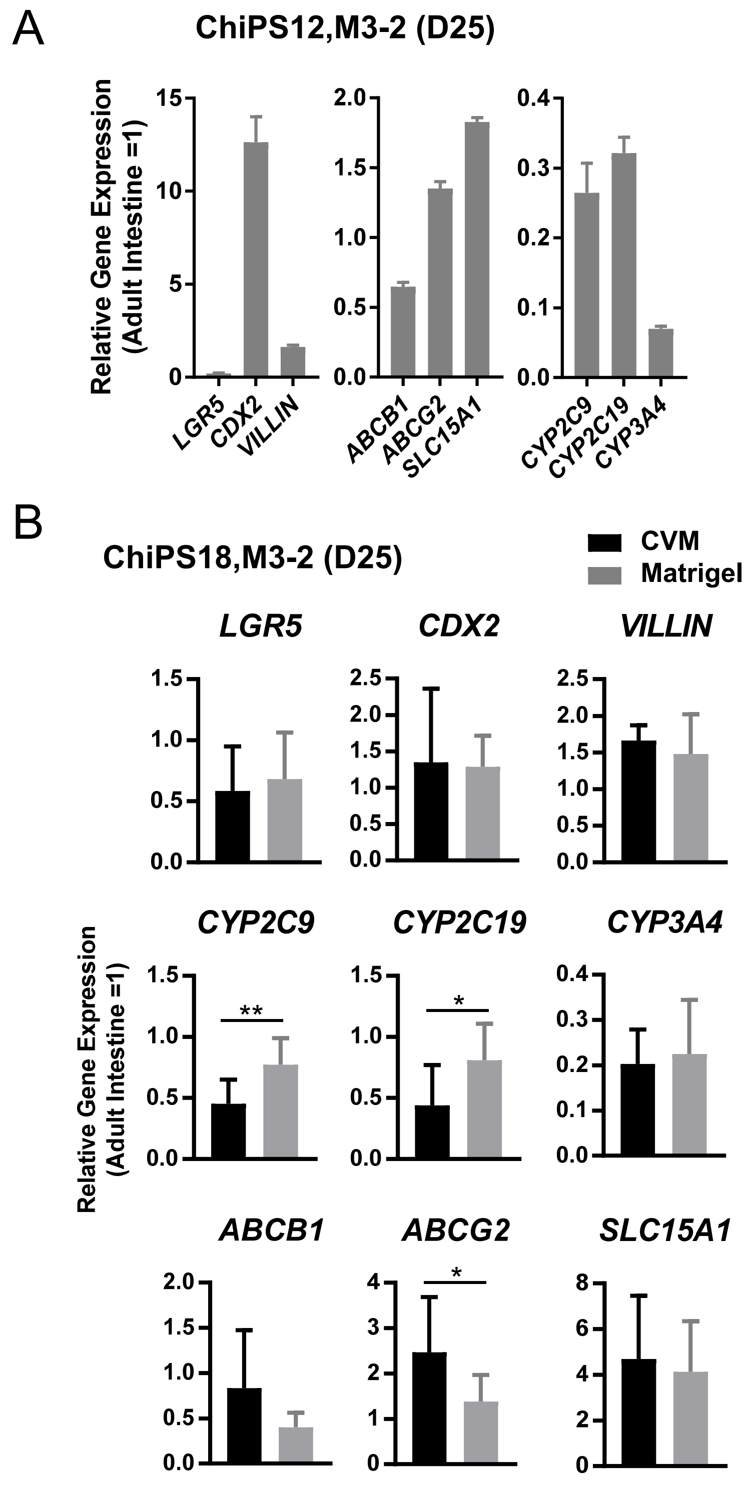

Figure S3 Intestinal differentiation of ChiPS12 cells and the comparison between CVM and Matrigel as scaffolds. Related to Figure 3.

Relative expression levels of intestinal differentiation markers in iPSC-derived enterocyte-like cells cultured using M3-2 media assayed on day 25 are shown. (A) ChiPS12-derived enterocyte-like cells grown on CVM. (B) ChiPS18-derived enterocyte-like cells grown on Matrigel (gray bars) expressed intestinal marker genes, although the expression levels were lower than those grown on CVM (black bars). Data are shown as the mean ± S.D. (n=3, independent experiments). Differences between groups were analyzed by Student's *t*-test, $^{*}p < 0.05$, $^{**}p < 0.01$.

Supplemental Experimental Procedure

Chemicals

Elacridar, verapamil, and midazolam were purchased from Wako Pure Chemical (Tokyo, Japan). [^{3}H]-digoxin, [^{3}H]-prazosin, [^{3}H]-propranolol and [^{3}H]-mannitol were purchased from PerkinElmer Life Sciences (Boston, MA, USA). Digoxin, testosterone, ketoconazole, 6β-hydroxytestosterone, diclofenac, ranitidine, famotidine, sulpiride, nadolol, and sulfasalazine were purchased from Sigma-Aldrich (St. louis, MO, USA). Antipyrine, metoprolol, hydrochlorothiazide, and fexofenadine were purchased from Wako Pure Chemical. Atenolol was purchased from LKT Laboratories (St. Paul, MN, USA). Acyclovir was purchased from Tokyo Chemical Industry (Tokyo, Japan).

Preparation of collagen vitrigel membrane chambers

Collagen xerogel membrane is manufactured by Kanto Chemical Co., Inc. (Tokyo, Japan) as described (Nakai et al., 2019).

Differentiation of iPS cells into intestinal cells

Undifferentiated ChiPS12, ChiPS18, or RPChiPS771 cells were first differentiated into the definitive endoderm (DE) on M15 feeder cells. Briefly, 100 mm diameter plates were pre-coated with mitomycin treated frozen M15 feeder cells at a density of 5×10^6 cells/dish. For definitive endoderm differentiation, undifferentiated ChiPS12, ChiPS18 or RPChiPS771 cells were plated onto M15 cell-coated 100 mm diameter plates at a density of $5{\times}10^5$ cells/dish and cultured in endoderm differentiation medium M1 supplemented with 3μM CHIR99021 (Wako) for 1 day, then changed to medium M1 without CHIR99021 and cultured for another 2 days. M1 consists of DMEM (ThermoFisher, Waltham, MA, USA, 11995-073) 4,500 mg/L glucose, Non-essential amino acids (NEAA; ThermoFisher, 11140050), L-glutamine

(Gln; Nacalai Tesque, Kyoto, Japan, 16948-04), penicillin-streptomycin (PS; Nacalai Tesque, 26252-94), 0.1mM β-mercaptoethanol (β-ME, Sigma-Aldrich), serum-free B27 supplement (ThermoFisher, 17504044), 100 ng/mL recombinant human activin A (Cell Guidance Systems, Cambridge, UK, GFH6). On day 3 (D3), ChiPS12, ChiPS18 or RPChiPS771–derived DE cells were dissociated, either plated directly for further differentiation, or frozen at 2.0×10^6 cells /ml in Bambanker hRM (NIPPON Genetics, Tokyo, Japan, CS-07-001) or STEM-CELLBANKER (Takara Bio, Kusatsu, Japan, CB045), and stocked in liquid N_2 until further use.

For intestine differentiation, D3 DE living cells or cryopreserved D3 DE cells were freeze-thawed and plated onto rehydrated vitrigel membrane (CVM) 24 well inserts (ad-MED Vitrigel™ 2, Kanto Chemical Co., Inc., culture area: 0.33 cm^2/insert), at a concentration of 8×10^4 cells/well in Thawing Medium. The volumes of the medium were 200 μL for the upper layer and 500 μL for the lower layer of the inserts. The media used for differentiation were: M2 for day (D) 4-D15, then changed to M3 (M3-0, M3-1 or M3-2) for D15-D21, or up to D40. Thawing Medium consists of DMEM, supplemented with 10% Fetal Bovine Serum (FBS; Hyclone, Logan, UT), 1% Insulin Transferrin Selenium (ITS; ThermoFisher, 41400-045), 10 μM Y27632 (WAKO, 251-00514), NEAA, L-Gln, PS, β-ME. M2 consists of DMEM (ThermoFisher, 11885-084, low glucose), supplemented with NEAA, L-Gln, PS, β-ME, D-Glucose (final concentration: 2000 mg/L), 10% KnockOut™ Serum Replacement (KSR; ThermoFisher, 10828028), 5 μM 6-Bromoindirubin-3'-oxime (BIO; WAKO, 029-16241) and 10 μM 3,5-difluorophenylacetyl)-L-alanyl-L-2-phenylglycine *tert*-butyl ester (DAPT; WAKO, 049-33583). M3-0 consists of Cellartis® Hepatocyte Maintenance Medium (MM) (Takara Bio, Y30051). Media in both upper and lower layers were replaced every 2 days with fresh medium and growth factors. M3-1 medium consists of William's E medium (ThermoFisher, A1217601) supplemented with L-Glu, HCM SingleQuots (without GA1000 and human Epithelial growth factor (EGF) (Lonza, Basel, Switzerland, CC-4182), PS (Nacalai tesque), 10 ng/ml recombinant human Hepatocyte growth factor (HGF; PeproTech, Rocky hill, NJ, USA, 100-39), 0.1 μM Dexamethasone (Sigma-Aldrich,

D8893), 1.4 μM BIO and 1μM 1α,25- dihydroxy vitamin D3 (VD3; Wako, 034-24921). M3-2 medium contains the same components as M3-1 medium except that 0.5% dimethyl sulfoxide (DMSO; Sigma-Aldrich, D2650) is used instead of HGF.

Alternatively, D3DE cells were plated onto 100 mm diameter normal tissue culture plates at a density of 4×10^6 cells/dish using the Thawing Medium supplemented with iMatrix-551 silk (Matrixome, Osaka, Japan, 892-021) at a concentration of 0.25 $\mu g/cm^2$ and changed to M2 medium next day (D4), and then cultured until day 10 (D10) with medium replaced every 2 days with fresh M2 medium. Then the cells were collected as D10 intestinal progenitor cells. D10 intestinal progenitor cells were cryopreserved for further use, or directly used for plating. D10 cells were plated onto rehydrated CVM 24 well inserts in M2 medium, at a concentration of 1.6×10^5 cells/well. 10 μM Y-27632 was added in the medium upon plating for the first 2 days. The cells were cultured in M2 until D15. The volume of the media was 200 μL for the upper layer and 500 μL for the lower layer of the inserts. The media were changed to M3-1 or M3-2 for D15-D21, or up to D30. The media were replaced every 2 days with fresh media. For differentiation on Matrigel, ChiPS18 D10 cells were passaged on Matrigel (BD Matrigel matrix, BD Biosciences, Bedford, MA, U.S.A. 356238)-coated 24-well cell culture inserts at a concentration of 1.6×10^5 cells/well and cultured in M2 until D15. The medium was changed to M3-2 for D15-D25.

Caco-2 cell culture

Caco-2 cells were cultured in DMEM medium (low glucose) supplemented with 10% FBS. For measurements of membrane integrities, Caco-2 cells were passaged onto CVM 24 well inserts at $5.0x10^4$ cells/well and continued cultures. Caco-2 cells typically formed monolayers approximately after cultured for seven days.

Immunocytochemistry

Cells were fixed in 4% paraformaldehyde (Nacalai Tesque) in PBS, permeabilized with 0.1% Triton X-100 (Nacalai Tesque). The following antibodies were used: anti-CDX2 (1:100, BioGenex, San Ramon, CA, MU392A-UC), anti-VILLIN (1:100, BD Transduction Laboratories, San Diego, 610359), Alexa 568-conjugated, and Alexa 488-conjugated antibodies (1:1000, ThermoFisher).

Real-time PCR analysis

RNA was extracted from iPS cells using the RNeasy micro-kit or QIAzol (Qiagen, Hilden, Germany) and then treated with DNase (Qiagen). For reverse transcription reactions, 2.5 μg RNA was reverse-transcribed using PrimeScript™ RT Master Mix (Takara Bio). For real-time PCR analysis, the mRNA expression was quantified with either TaqMan Primers and Probe or using SYBR Green on a StepOne Plus (Applied Biosystems, Foster City, CA, USA). β-ACTIN and GAPDH were used as internal controls. Target mRNA levels were expressed as fold-change against human adult small intestine (ASI; ASI=1). Primer details are listed in Supplemental Table S2.

TEER measurement

Membrane integrities of human iPSCs derived intestine cells and Caco-2 cells cultured on CVM were measured after the medium change, using a Millicell ERS-2 (Epithelial Volt-Ohm Meter, Millipore).

Transcellular transport assays and measurement of CYP3A metabolite

In Figure 2 and Figure S1, for assessing the transporter activity of P-gp, time-dependent directional (B-to-

A and A-to-B) transport of [^{3}H]-digoxin (0.1μCi/3mL; specific activity: 26.3Ci/mmol) across cell monolayer was measured in the absence or presence of 100 μM verapamil. For assessing BCRP and P-gp activities, time-dependent directional transport of [^{3}H]-prazosin (0.1 μCi/3mL; specific activity: 85.3Ci/mmol) was measured in the absence or presence of 20 μM elacridar. For assessing transcellular transport mediated by passive membrane permeation and paracellular transport across cell monolayer, [^{3}H]-propranolol (0.1 μCi/3mL; specific activity: 25.0Ci/mmol) and [^{3}H]-mannitol (0.1 μCi/3mL; specific activity: 24.7Ci/mmol) were tested, respectively.

For assaying the transport of the above substrates, the culture medium for hiPS-derived enterocyte-like cells was removed and replaced with transport buffer (TB; 118 mM NaCl, 23.8 mM $NaHCO_3$, 4.8 mM KCl, 1.0 mM KH_2PO_4, 1.2 mM $MgSO_4$, 12.5 mM HEPES, 5 mM glucose and 1.5 mM $CaCl_2$ adjusted at pH7.4) at 37°C, and pre-incubated for 10 min. Assays were started by replacing the TB with TB containing substrates +/- inhibitor. Volumes of TB added were 200 μl to the upper chamber and 500 μl to the lower chamber of the CVM insert. Then, 30, 60, and 120 min after starting the drug incubation, aliquots of the medium at the opposite compartment of initial drug application were sampled and replaced with the same sampled volume of fresh TB (substrates +/- inhibitors). For the measurement of A-to-B transport, 100 μl of TB was sampled from the basal compartment, while for the measurement of B-to-A transport, 50 μl of TB was sampled from the apical compartment. Then, samples were mixed with CLEAR-SOL I (Nacalai Tesque), and their radioactivity was quantified with a liquid scintillation counter (PerkinElmer). In parallel, CYP3A-mediated metabolism of midazolam (10 μM) for 2 hours at 37°C was observed by measuring the formation of major metabolite, 1'-OH midazolam with LC-MS/MS (Shimadzu Prominence Ultra-Fast Liquid Chromatography/ABSciex QTRAP 5500).

Alkaline phosphatase activity measurement

The hiPS-derived enterocyte-like cells were fixed and stained for alkaline phosphatase activity using a StemTAG Alkaline phosphatase staining and activity assay kit (Cell Biolabs, Inc, San Diego, CA, USA), according to the manufacturer's protocol.

Permeability measurements

The apparent permeability coefficient (P_{app}) for digoxin in Figure S2 or each of the 15 compounds in Figure 5 was determined as follows: the culture medium for hiPS-derived enterocyte-like cells was replaced with 5% FBS supplemented Hanks' Balanced Salt Solutions (HBSS; ThermoFisher, 4025092). Assays were started by replacing the FBS-containing HBSS buffer with substrate +/- inhibitor and incubated at 37°C for 2 h. The unlabeled compounds were analyzed by LC-MS/MS. The LC-MS/MS system consisted of a Waters ACQUITY UPLC (Waters Corporation, Milford, MA, USA) and a Waters Quattro Ultima mass spectrometer (Waters Corporation). The multiple reaction monitoring modes were used to monitor ions. The detailed conditions for the analyses of the compounds are shown in Supplemental Table S1. The apparent membrane permeability coefficient (P_{app}) was calculated as follows. $Papp = \frac{dQ}{dt} \times \frac{1}{AC_0}$

Where dQ/dt is the amount of the compound permeated per unit of time, A is the surface area of insert membrane (0.33 cm^2), and C_0 is the initial compound concentration in the donor chamber. Differentiated cells showing TEER >30 Ω・cm^2 were used. The relationship between P_{app} values and fraction of an oral dose absorbed from the intestinal lumen (Fa (%)) values in humans obtained from literature was represented as the following theoretical model according to the previous report (Amidon et al., 1988). $\mathrm{Fa} = 100 \times \left(1 - exp\left(-\alpha \times P_{app}\right)\right)$. Where α is the scaling factor. The fitting curve was calculated by nonlinear regression using Phoenix WinNonlin (Ver. 8.1, Certara, L.P.).

Measurement for CYP metabolites

In Figure S2B, hiPSC-derived enterocyte culture medium (M3-0) was removed. In Figure. 4, hiPSC-derived enterocyte culture medium (M3-2) was removed. The culture media were replaced with transport buffer (TB) containing substrates (midazolam 20 μM or testosterone 50 μM) at 37°C, and pre-incubated for 10 min. Assays were started by adding TB containing midazolam or testosterone, with or without a potent CYP3A4 inhibitor, ketoconazole (Sigma-Aldrich) at 0.5 or 5 μM, to both the apical (200 μl) and basal (500 μl) compartments. After 120 min incubation, all the incubation media were collected from both upper and lower compartments and samples were kept at -80°C until performing LC-MS/MS analysis of the metabolite of midazolam, 1'-OH midazolam or 6β-OH testosterone, respectively (detailed analysis conditions are listed in Supplemental Table S1). The protein amount per well was quantified using the Pierce BCA protein assay kit (ThermoFisher) according to the manufacturer's instructions. Metabolic clearance was normalized with the cellular protein amount. Since the amounts of metabolites were found to reach a plateau at 30 min, the values obtained at 30 min were used to calculate the metabolite rate in Figure 4E.

Compound	Instrument (LC, MS)	Column	LC condition: Mobile phase A	LC condition: Mobile phase B	LC condition: Gradient condition % B/min	LC condition: Flow rate mL / min	MS condition: cone voltage V	MS condition: collision energy eV	MS condition: Monitoring ion (m / z) precursor	MS condition: Monitoring ion (m / z) product
antipyrine	1, 1	1	0.1% HCOOH in water	acetonitrile	20-95-95-20 /0-0.50-0.90-0.91-1.10	1.0	12	51	189.1	56.1
propranolol	1, 1	1	0.1% HCOOH in water	acetonitrile	20-95-95-20 /0-0.50-0.90-0.91-1.10	1.0	100	25	260.2	183.1
metoprolol	1, 1	1	0.1% HCOOH in water	acetonitrile	20-95-95-20 /0-0.50-0.90-0.91-1.10	1.0	40	25	268.1	116.0
diclofenac	1, 1	1	0.1% HCOOH in water	acetonitrile	20-95-95-20 /0-0.50-0.90-0.91-1.10	1.0	34	50	296.0	214.1
digoxin	1, 1	1	0.1% HCOOH in water	acetonitrile	20-95-95-20 /0-0.50-0.90-0.91-1.10	1.0	194	14<br>22	781.6<br>781.6	651.4<br>243.0
hydrochlorothiazide	1, 1	1	0.1% HCOOH in water	acetonitrile	5-95-95-5 /0-0.50-0.70-0.71-0.9	1.0	-88	-27	295.9	268.9
atenolol	1, 1	1	0.1% HCOOH in water	acetonitrile	2-50-50-2 /0-0.50-0.70-0.71-0.9	1.0	51	26	267.2	190.1
famotidine	1, 1	1	0.1% HCOOH in water	acetonitrile	2-50-50-2 /0-0.50-0.70-0.71-0.9	1.0	32	27	338.0	189.0
sulpiride	1, 1	1	0.1% HCOOH in water	acetonitrile	2-50-50-2 /0-0.50-0.70-0.71-0.9	1.0	52	34	342.1	112.1
nadolol	1, 1	1	0.1% HCOOH in water	acetonitrile	2-50-50-2 /0-0.50-0.70-0.71-0.9	1.0	79	23	310.1	254.1
acyclovir	1, 1	1	0.1% HCOOH in water	acetonitrile	2-50-50-2 /0-0.50-0.70-0.71-0.9	1.0	27	18	226.0	152.0
ranitidine	1, 1	1	0.1% HCOOH in water	acetonitrile	5-95-95-5 /0-0.90-1.20-1.21-1.50	1.0	1	23	315.1	176.0
sulfasalazine	1, 1	1	0.1% HCOOH in water	acetonitrile	5-95-95-5 /0-0.90-1.20-1.21-1.50	1.0	84	40	399.1	223.1
fexofenadine	1, 1	1	0.1% HCOOH in water	acetonitrile	5-95-95-5 /0-0.90-1.20-1.21-1.50	1.0	112	38	502.3	466.3
testosterone	1, 1	1	0.1% HCOOH in water	acetonitrile	50-60-60-95-95-50 /0-0.40-0.90-0.91-1.1-1.11-1.4	1.0	80	28	289.1	97.0
6beta-hydroxytestosterone	1, 1	1	0.1% HCOOH in water	acetonitrile	30-50-50-95-95-30 /0-0.40-0.60-0.61-0.8-0.81-1.0	1.0	84	72	305.1	91.0
midazolam	1, 1	1	0.1% HCOOH in water	acetonitrile	30-50-50-95-95-30 /0-0.40-0.50-0.51-0.70-0.71-0.90	1.0	7	38	325.6	291.2
1-hydroxy midazolam	1, 1	1	0.1% HCOOH in water	acetonitrile	30-50-50-95-95-30 /0-0.40-0.50-0.51-0.70-0.71-0.90	1.0	130	37	341.6	202.8

Supplemental Table S1. LC-MS/MS conditions for analysis

The compounds were determined using LC-MS/MS. The LC-MS/MS system consisted of a Nexera UHPLC (Shimadzu, Kyoto, LC instrument 1) and a Triple Quad 6500 Plus system (AB SCIEX, Tokyo, MS instrument 1) or a Triple Quad 6500 system (AB SCIEX, Tokyo, MS instrument 2). The multiple reaction monitoring mode was used to monitor ions. The columns for chromatographic separation of these analytes were CAPCELL PAK ADME (2.1 mm I.D. × 50 mm, 3 µm, OSAKA SODA, Osaka, column 1) and CAPCELL PAK ADME (2.1 mm I.D. × 20 mm, 3 µm, OSAKA SODA, Osaka, column 2) used.

Supplemental Table S2. Primer sequences, or TaqMan primers and probe ID of the genes used in real-time PCR

A) Primer sequences or TaqMan probe ID used in Figure 1D-F.

Gene Symbol	Forward Primer Sequence	Reverse Primer Sequence
GAPDH	CGAGATCCCTCCAAAATCAA	CATGAGTCCTTCCACGATACCAA
CDX2	GAGGGGGTGGTTATTGGACT	AGGAAGTCCAGGTTGGCTCT
LGR5	CTCTTCCTCAAACCGTCTGC	GCAACTGCTGGAAAGTGTCA
ABCB1	CTTATGCTCTGGCCTTCTGG	GGAGATGCCTGTCCAACACT
ABCG2	TTAAGTGGAAACTGCTGCTTTAGAGT	TCGGTCTTAACCAAAGGCTCA
SLC15A1	GCAATATCATTGTGCTCATCGT	CAATCTCTGCTGGGTTGATGT

Gene Symbol	TaqMan Primers and Probe ID*
GAPDH	Hs02758991_g1
VILLIN	Hs01031724_m1
CYP2C9	Hs00426397_m1
CYP2C19	Hs00426380_m1
CYP3A4	Hs00430021_m1

*ABI Expression Assay

B) Primer sequences used in Figure 1, 3, 4 and S3.

Gene Symbol	Forward Primer Sequence	Reverse Primer Sequence
LGR5	TAACTGGAACTGCAAACCTGGAGA	CTGATTGCAGACGGTTTGAGGA
CDX2	TCACTGGGCATTTCCGTGAG	GTGGATCGGCCAGATAACAAGA
VILLIN	CGACTGCTACCTGCTGCTCTACAC	CGGCTTGATAAGCTGATGCTGTAA
CYP2B6	CCAGCTTCCGAGGGTACATCA	TTCAAAGTAGTGTGGGTCATGGAGA
CYP2C9	AACACTGCAGTTGACTTGTTTGGAG	GGTTTCTGCCAATCACACGTTC
CYP2C19	AATCACTGCAGCTGACTTACTTGGA	CCGGTTTCTGCCAATGACAC
CYP3A4	GAAACACAGATCCCCCTGAA	CTGGTGTTCTCAGGCACAGA
CYP3A7	AAGGTCGCCTCAAAGAGACA	TGCACTTTCTGCTGGACATC
UGT1A1	TGGCTGTTCCCACTTACTGCAC	AGGGTCCGTCAGCATGACATC
ABCB1	GGAGCCTACTTGGTGGCACATAA	TGGCATAGTCAGGAGCAAATGAAC
ABCG2	CATGGTGTATAGACGCCCTGAC	GTTCCCAAATTGATGTTGTGACAGA
SLC15A1	TCACCTGTGGCGAAGTGGTC	AGCAGCCATCCTGCCTGAA
ALPI	CATTCCAGGTCACCAGATCCA	AGAAATCTATGCCCAGCATCCAG
ACTB	TGGCACCCAGCACAATGAA	CTAAGTCATAGTCCGCCTAGAAGCA
GAPDH	GCACCGTCAAGGCTGAGAAC	TGGTGAAGACGCCAGTGGA
